# Dietary assessment of type 2 diabetic patients using healthful plant-based diet score in the Eastern Province of Saudi Arabia

**DOI:** 10.1186/s40795-024-00843-z

**Published:** 2024-02-28

**Authors:** Rudaynah A. Alali, Suad A. Alateeq, Afnan F. Almuhanna, Abdulmohsen H. Al Elq, Waleed I. Albaker, Alawi Habara, Fatima A. Alrubaish, Chittibabu Vatte, Bao-Li Loza, Fahad A. Al-Muhanna, Amein K. Al-Ali

**Affiliations:** 1 Department of Internal Medicine, King Fahad Hospital of the University, Al Khobar, Saudi Arabia; 2 Imam Abdulrahman Bin Faisal University, 31441 Dammam, Saudi Arabia; 3 Department of Clinical Biochemistry College of Medicine, Imam Abdulrahman Bin Faisal University, 31441 Dammam, Saudi Arabia; 4 Department of Radiology, King Fahad Hospital of the University, Al Khobar, Saudi Arabia; 5 Department of Surgery, Perelman School of Medicine, University of Pennsylvania, 19104 Pennsylvania, PA USA

**Keywords:** Dietary patterns, T2D, Nutrition, Glycated hemoglobin (HbA1c), Glycemic Control, Lipid Profile

## Abstract

**Background:**

Diabetes mellitus is a chronic disease characterized by a wide range of metabolic problems. The current study sought to assess nutritional habits of Saudi patients with type 2 diabetes (T2D) and to propose recommendations to improve these patients’ dietary habits and delay possible disease complications.

**Methods:**

Over a period of three years, (2017–2019) 577 patients with T2D attending the outpatient’s diabetic clinics at King Fahd Hospital of the University, Al Khobar, Saudi Arabia were invited to participate in this study. Data of dietary intake were collected by trained nurses using a pretested structured validated semi quantitative food frequency questionnaire. The dietary data were collected using 7-day dietary recall questionnaire. A modified score system that associates dietary habits with glycemic control and lipid profile was used.

**Results:**

Overall, a high healthful plant-based diet score was associated with a significant (*P* = 0.018) reduction in triglycerides (TG) level (mean difference − 3.78%; 95% CI, -0.65% to -6.81%) and a statistically non-significant (*P* = 0.06) increase in high density lipoprotein (HDL) levels (mean difference 1.87%; 95% CI -0.06–3.84%) in T2D patients from the Eastern Province of Saudi Arabia. Additionally, in our patient group, the prevalence of coronary artery disease, stroke, peripheral artery disease, and chronic kidney disease in T2D patients was 11.3%, 6.2%, 3.3%, and 8.4%, respectively and were higher when compared to the prevalence in the general population.

**Conclusion:**

The present study showed that adherence to a healthful plant-based diet, when compared to high glycemic index diet, is associated with a favorable outcome in glycemic control and lipid profile in T2D patients. Prior assessment of total diet quality may be beneficial when giving nutritional advice to T2D patients with the possibility of improving glycemic control and lipid profile.

**Supplementary Information:**

The online version contains supplementary material available at 10.1186/s40795-024-00843-z.

## Introduction

Type 2 diabetes mellitus (T2D) is a chronic metabolic disorder that is becoming a growing public health problem [1–2]. Approximately 1 in 11 adults worldwide have diabetes mellitus (DM) with T2D being the most prevalent type, constituting 90% of all diabetes mellitus (DM individuals [3]. If T2D patients maintain a good glycemic index, it will delay or prevent secondary complications such as cardiovscular disese, chronic kidney disease (CKD), and stroke [4–6]. Therefore, it is not surprising that DM can lead to a reduction in the quality of life and lifespan of affected individuals [7].

In Saudi Arabia, the prevalence of DM in 2021 was reported to be 18.7% and is expected to rise to 20.4% by 2030 with 17% of T2D individuals having coronary artery diseases (CAD) [8–9]. A recent study conducted in the Eastern Province of Saudi Arabia reported variations in the health status of T2D patients, ranging from a perfect health state to a state with severe disease complications. The rapid rate of increase of T2D disease in some areas of Saudi Arabia, which increased from 16% in 2005 to over 25% in 2011, is thought to be due to rapid lifestyle changes such as diet and sedentary lifestyle, as well as adverse environmental factors [10].

An unhealthy diet is one of the major contributors toward increasing the risk of disease development globally, including T2D [3, 11]. A study conducted in the United States reported that the leading cause of death and the third leading cause of disability-adjusted life-year loss was an inadequate poor diet. A poor diet refers to the quantity of food, the source of food, and the macronutrients, which are all associated with the disease risk. Macronutrients can be affected by fluctuations in the economy, nutrition-related policies and the methods used in food processing, all of which affect the composition of the macronutrients and the quality of food available to the population.

Consequently, evaluation of the dietary habits of T2D patients is important to recognize challenges that need to be overcome to improve the dietary habits of T2D populations. In patients with DM, medical nutrition treatment (MNT) improves metabolic outcomes such as glycated hemoglobin (HbA1c). For patients with T2D, MNT administered by a qualified dietician is linked with a 0.5–2% reduction in HbA1c [4, 11]. One study in the region has shown that dietary intervention in T2D patients was associated with improved glycemic index, reflected by lower level of HbA1c [12]. However, cultural and socioeconomic variables impact people’s dietary habits and choices, and studies have revealed ethnic variances in glycemic response to common foods, thereby emphasizing the need to take ethnic differences into account when formulating dietary recommendations [13].

Although the Healthful Plant-based Diet Index (HPDI) was developed to examine whether Americans were following the Dietary Guidelines for Americans, it is now widely used to assess diet quality in other countries and to compare diet quality and health consequences [14]. Minimal community based epidemiological studies have been conducted on Saudi Arab diabetic populations to date. Both screening and therapies have not caught up with the benefits of new technologies which have advanced in disease treatment. Furthermore, uncontrolled diabetes is a significant health and financial burden on the health institutes in Saudi Arabia, and it is on an increasing trend (8–9). In this context, and as data on Saudi dietary habits are scarce, the current study sought to assess nutritional habits of a large sample size of Saudi subjects with clinically diagnosed T2D in order to illustrate the effects of unhealthy dietary habits on the glycemic index and lipid profile in these patients and its relationship to disease complications. In addition, we have proposed recommendations to improve the glycemic control and lipid profile in T2D patients which may consequently have a positive effect on reducing or delaying possible disease complications.

## Methods

### Study population

Over a period of three years, (2017–2019) a prospective cohort of patients with T2D (*N* = 577) were randomly recruited from the outpatient diabetic clinics at King Fahd Hospital of the University, Al Khobar, Saudi Arabia and were invited to participate in this study. A study summary in Arabic, which explained the purpose and exact nature of the study, was given to each recruited patient. The study was approved by Imam Abdulrahman Bin Faisal University’s Institutional Review Board committee, and each participant signed a written informed consent form.

### Inclusion & exclusion criteria

The inclusion criteria for this study were Saudi nationals aged 18 years and over with a confirmed clinical diagnosis of T2D according to the WHO criteria and definitions. All T2D subjects with a co-existing malignant disease, as confirmed by the medical records, were excluded from the study.

### Study design

Dietary intake data were collected by a trained nurses using a pretested 7-day semi-quantitative food frequency questionnaire (7-d SQFFQ) to capture dietary intake of the participants over a period of one week preceding the interview. Participants were asked how often, on average, they had consumed a standard portion size of each food in the preceding week. Where applicable, visual aids were used to assist the participants in assessing the amounts of each principal food item included on the questionnaire. The data of the main study were collected by two native Arabic speaking interviewees who interviewed the same participant on two separate occasions to recall the dietary intake for the preceding week. The data from the two readings were combined and a mean reading was recorded for that participant.

Socio-demographic data, biochemical parameters (including HbA1c and fasting glucose) and comorbidities were collected from the patients’ medical records. Fasting blood glucose level and HbA1c were determined according to normal procedures on the day of the first interview.

Using the food components and the frequencies consumed, we calculated the HPDI, which has been evaluated in large studies for its association with coronary heart disease, to measure participants’ dietary patterns [15]. The healthy plant foods were scored positively, while less healthy animal-derived foods were scored negatively.

### Questionnaire

The 7-d SQFFQ utilized in this study was developed by Yuan et al. (2017) and has been validated among men and women in Women’s Lifestyle Validation Study (2010–2012) [16–17]. Furthermore, the results of the Yuan et al. (2017) studies have offered statistically acceptable estimates of the various food intakes which have since been validated in many populations.

The questionnaire used in this study was modified to incorporate the most common food patterns and food items consumed by the local population. The 7-d SQFFQ used 46 of the most consumed food items in this population. These food items were grouped as oils, dairy products, fruit and vegetables, meat, poultry, fish, bread, breakfast cereals, carbohydrate-rich foods, beverages, and some processed foods. The oils included margarine, butter, and vegetable oil. Dairy products included milk (full fat, low fat and skimmed) in addition to cheese and yoghurt. Fruit and vegetables included tinned fruit and fresh peaches, plums, grapes, melon, bananas, apples, and oranges, while vegetables mainly included salad vegetables. Meat, poultry, and fish included lamb, beef, chicken, and fish but focused on the cooking method of each (stewed, minced, grilled, and fried). Bread included brown and white bread while breakfast cereals included among other items, porridge and cornflakes. The carbohydrate-rich foods included rice, potatoes, and pasta while the processed foods included chocolate and cake. Beverages included juice and cola and sweetened coffee and tea. The questionnaire was translated into Arabic by a sub-committee composed of physicians, nurses, and nutritionists who were fluent in both Arabic and English. The validation of the Arabic version of the questionnaire was conducted by asking two interviewees (trained nurses) to interview the same 15 patients independently and compare the results on the questionnaires to assess interrater reliability using κ coefficient. All participants were also interviewed by trained nurses for completion of the translated and validated questionnaire. From the patients’ responses, two plant diet indices were determined (healthful plant-based diet index [hPDI] and unhealthful plant-based diet index [uPDI]). A higher hPDI score indicated a higher consumption of a healthy plant diet while a higher unhealthful plant-based diet index (uPDI) indicated a lower consumption of a healthy plant diet. The components and scoring criteria were modified from the original hPDI to tailor to our specific study (Table 1 and Supplementary Table S1A). The modified hPDI included 15 components, with the total score potentially ranging from 15 to 72. The modified hPDI components and criteria for scoring are shown in Supplementary Table S1B.

**Table 1:** Modified Healthful Plant-based Diet Index components and criteria for scoring (based on data collected). The criteria for minimum score (least healthy) for example, in the healthy plant food group taking less vegetable (lowest quintile) is considered less healthy, while taking more vegetable (highest quintile) is healthiest. The same thing applies to unhealthy plant food group, for example sugar sweetened beverages, taking highest quintile is the criteria for least healthy while taking lowest quintile is considered healthy

Component	Criteria for minimum score of 1 (least healthy- higher uPDI)	Criteria for maximum score (healthiest-high hPDI)
**Healthy Plant Food Groups**		
Whole grains (Bread, brown)	Lowest quartile	Highest quartile
Fruits	Lowest quintile	Highest quintile
Vegetables (salad)	Lowest tertile	Highest tertile
Vegetable oils	Lowest quartile	Highest quartile
Tea & Coffee	Lowest quintile	Highest quintile
**Unhealthy Plant Food Groups**		
Fruit juices	Highest quintile	Lowest quintile
Refined grains	Highest quintile	Lowest quintile
Potatoes	Highest quintile	Lowest quintile
Beverages (juice, cola and sweetened coffee and tea)	Highest quintile	Lowest quintile
Sweets and desserts (chocolate, cake)	Highest quartile	Lowest quartile
**Animal Food Groups**		
Animal fat (Butter)	Highest quintile	Lowest quintile
Dairy	Highest quintile	Lowest quintile
Egg	NA	NA
Fish or seafood	Highest quintile	Lowest quintile
Meat	Highest quintile	Lowest quintile
Miscellaneous animal-based foods	NA	NA
Trans Fat (Margarine)	Highest quintile	Lowest quintile

### Statistical analysis

We performed multiple regression to test the association of the continuous outcome phenotypes (in this case, A1c and lipid profile) with serving frequencies of various food items/types (as continuous variables), while adjusting for participants’ age and sex. We performed multiple regression to test the association of the continuous outcome phenotypes (in this case, A1c and lipid profile) with serving frequencies of various food items/types (as continuous variables), while adjusting for participants’ age and sex. T-statistics were used to test the significance of the regression slopes/coefficients, with the null hypothesis being the slope/coefficient equal to zero, and alternative hypothesis being the slope/coefficient not equal to zero.

The associations between binary outcomes (CAD, stroke, peripheral artery disease (PAD), CKD and hPDI or individual components of hPDI were tested using multivariable logistic regression, adjusting for participants’ age and sex. The association of HbA1c, total cholesterol (TC), triglycerides (TG), high density lipoprotein (HDL), and low-density lipoprotein (LDL) levels (as outcome variables) with hPDI or individual components of hPDI (as independent variables) were tested using multiple regression, adjusting for participants’ age and sex. All tests were two-sided. *P*-value < 0.05 was considered statistically significant. All statistical analyses were performed using R 4.2.2.

## Results

A total of 577 T2D patients were registered in the study with a female preponderance (57.2%). However, only 523 patients, aged 21–80 years, were included in the statistical analysis due to missing data for the excluded patients, Based on the power calculation of the most frequent comorbidity CAD (prevalence~10%), at alpha of 0.05, we anticipated to achieve an 80% power to detect a very modest OR of 1.50 with a sample size of 500 participants.

The mean duration of T2D since diagnosis was 11.5 ± 8.6 years. Mean fasting glucose level was 168.4 ± 73.5. Of the study population, 85% consumed meals on time (3 meals per day) while the remaining 15% consumed a meal more than 5 times per day. Chicken was consumed more frequently in 89.1% of patients followed by mutton in 77.9% of patients (Table 2). Full fat milk was consumed by 24% of patients while low fat milk was consumed by 33.7% of the patients followed by skimmed milk at 4.1%. Sweetened foods including cake, chocolate, juice, cola and sweetened tea was consumed by 69.4%, 56%, 71.2% 34.5% and 32.7% of patients, respectively (Supplementary Table S1A).

**Table 2:** Patient’s characteristics. Epidemiological and biochemical characterization of Saudi diabetic patients residing in the Eastern Province of the country

Variables		Percentage of Patients
Age of patients (mean ± SD) (Years)		52.8 ± 10.1
Mean duration since diagnosis (Years)		11.5 ± 8.6
Mean Fasting glucose concentration (mg/dL)		168.4 ± 73.5
Mean *HbA1c (%)		8.3 ± 1.9
Gender		N (%)
	Male	Full
	Female	330 (57.2)
Type of consumed fat		
	Margarine	107 (18.5)
	Butter	74 (12.8)
	Vegetable oil	514 (89.1)
Meat consumption		
	Beef	331 (57.3)
	Mutton	450 (77.9)
	Chicken	500 (86.6)
	Fish	380 (65.8)
Breakfast routine		
	White bread	337 (58.4)
	Brown bread	284 (49.2)
	Porridge	136 (23.6)
	Cornflakes	123 (21.3)

HbA1c: glycated hemoglobin

**Fig. 1:**
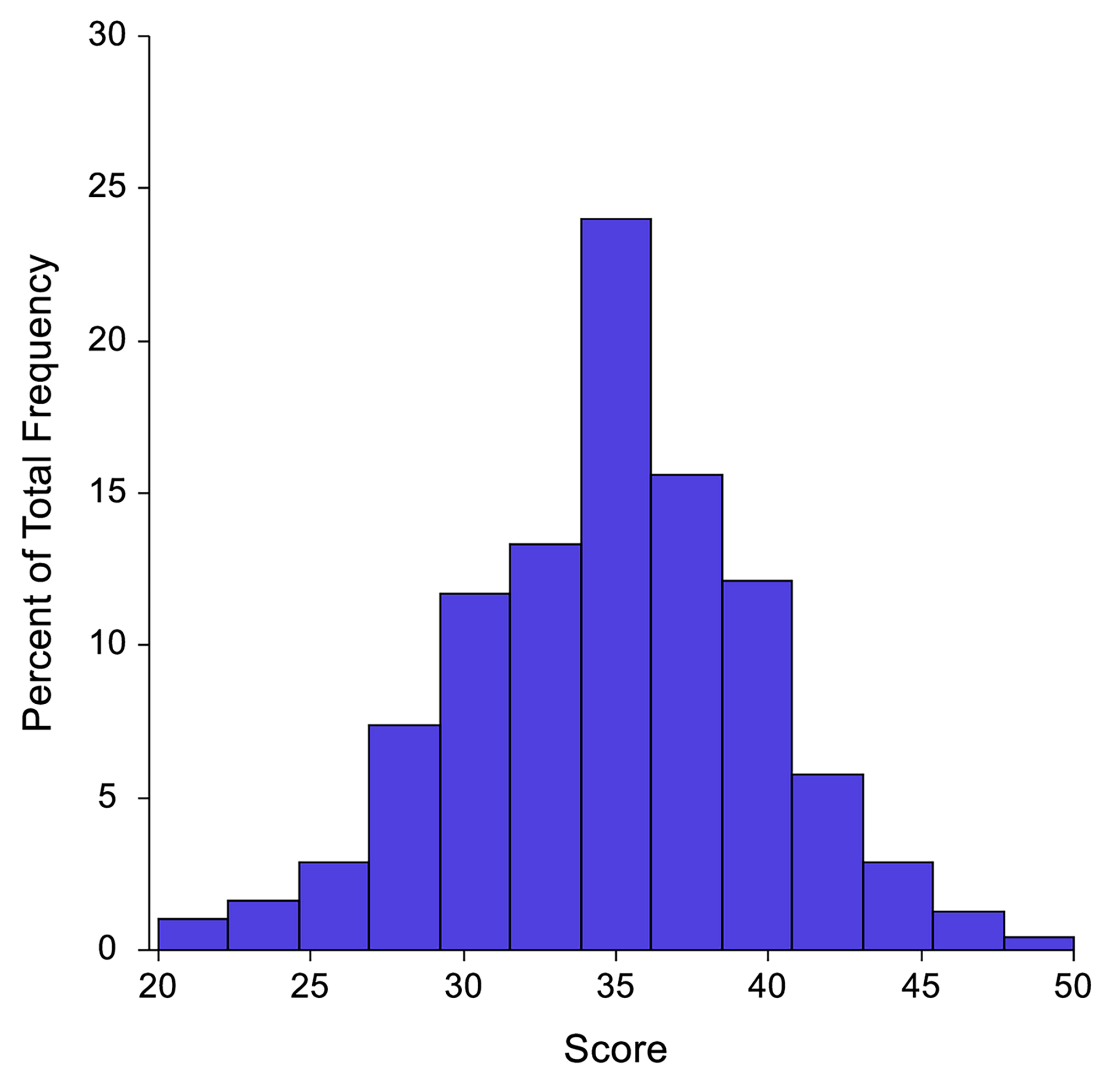
The distribution of the modified healthful plant-based diet score among patients with T2D. The Scores are normally distributed (bell-shape curve) with mean ± SD of 34.87 ± 4.96; min = 21, max = 48

Figure 1 shows the percentage distribution for patients with T2D within the modified healthful plant-based diet, and it shows a normal distribution with a mean ± SD of 34.87 ± 4.96. The modified hPDI scores for saturated fats (animal diet), meat, trans fats, refined grains, margarine, full fat dairy products and potatoes were in the highest quintile in the minimum score criteria, while whole grain, fruit, vegetable oils, tea and coffee were in the highest quintile in the maximum score criteria.

Table 3 shows the association between HbA1c, TC, TG, HDL and LDL with the modified healthful plants-based diet score. The mean HbA1c level in our cohort of T2D patients was similar to that reported by other investigators [18].

**Table 3:** Association of HbA1c, TC, TG, HDL, LDL^*^ with Modified Healthful Plant-based Diet Score^*^ (as Quartiles) Among DM Patients, Adjusting for Age and Sex. **(A)** All quintiles of modified healthful plant-based diet score. **(B)** Highest quintile vs. lowest quintiles of modified healthful plant-based diet score

Outcome Variable	Number of Patients	% Change (95%CI) in mean per increase in quartile	*P*-Value
HbA1c (%)	447	-0.02% (-1.42%– 1.39%)	0.98
TC (mg/dL)	468	-0.23% (-2.13%– 1.71%)	0.82
TG (mg/dL)	469	-3.78% (-0.65%– -6.81%)	0.018^#^
HDL (mg/dL)	468	1.87% (-0.06– 3.84%)	0.06
LDL (mg/dL)	468	-0.43% (-2.84%– 2.04%)	0.73
Outcome Variable	Number of Patients	% Change (95%CI) in mean per increase in quartile	*P*-Value
HbA1c (%)	176	0% (-6.67 − 7.15%)	0.85
TC (mg/dL)	189	0% (-8.8 − 12.2%)	0.85
TG (mg/dL)	190	-14.89% (-27.56% − 0%)	0.06
HDL (mg/dL)	189	7.15% (-2.28 − 17.49%)	0.13
LDL (mg/dL)	189	2.33% (-8.8 − 14.82%)	0.66

^*^ Scores were classified into quintiles. # indicates significance at *p* < 0.05. HbA1c: glycated hemoglobin, TC: total cholesterol, TG: triglycerides. HDL: high density lipoprotein, LDL: low density lipoprotein

**Fig. 2:**
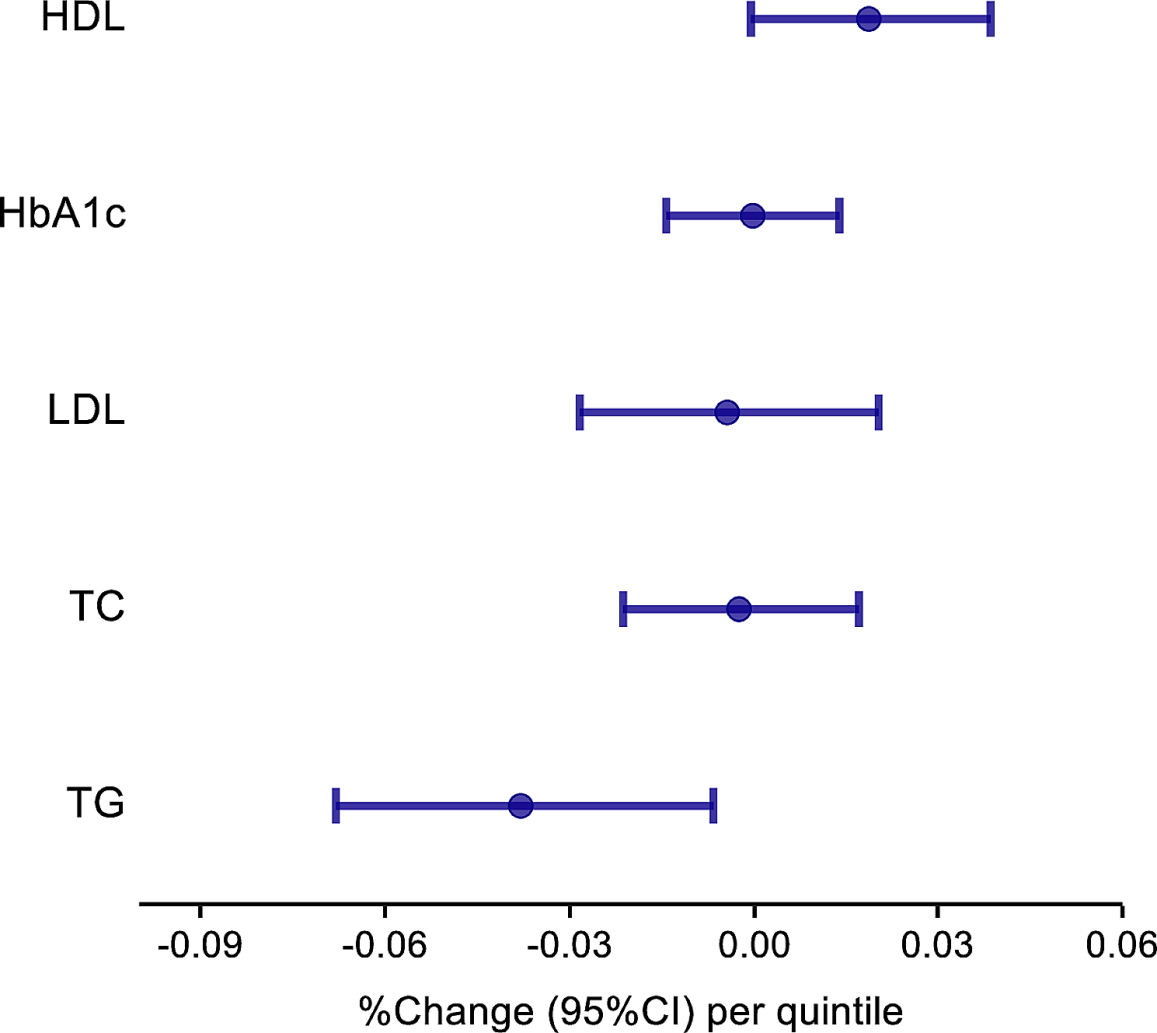
Forest plot representing the results shown in Table 3 for the association of HbA1c, TC, TG, HDL, LDL with Modified Healthful Plant-based Diet Score (as quintiles) among T2D patients, adjusting for age and sex

The association between HbA1c and lipid profile with healthier eating habits was as expected. Healthy eating appears to lead to a lower level of HbA1c (difference − 0.02%; 95% CI, -1.42–1.39%), TC (difference − 0.23%; 95% CI, -2.13–1.71%), TG (difference − 3.78%; 95% CI, -0.65% to -6.81%) and LDL (difference − 0.43%; 95% CI, -2.84–2.04%) as shown by the negative regression coefficient. However, only TG shows a strong significant (*P* = 0.018) association with the healthful plants-based diet score in T2D patients. Additionally, a higher HDL level (difference 1.87%; 95% CI -0.06–3.84%, positive regression coefficient) was observed with healthier eating habits with a *p*-value of 0.06. Figure 2 shows a forest plot representing the data from Table 3. Table 4 shows the association of HbA1c and lipid profile in T2D patients with some individual food categories. High butter consumption led to a significant (*P* = 0.03) increase in the level of HbA1c (difference 4.44%; 95% CI 0.53–8.5%). Consuming more fish in the diet led to a significant (*P* = 0.04) increase in LDL (difference 3%; 95% CI 0.18%– 5.9%). Consuming more whole grains in the form of brown bread and vegetables was significantly (*P* = 0.02; 0.01) associated with an increased HDL level (difference 3.69% and 4.2%, respectively with 95% CI 0.59–6.88% and 1.21–7.27%, respectively). Consuming fewer sugar sweetened beverages was also associated with a significant (*P* = 0.02) increase in HDL (difference 3.07%; 95% CI 0.49–5.71%). Additionally, drinking fewer sugar sweetened beverages was associated with significantly (*P* = 0.04) lower levels of TG (difference − 4.31%; 95% CI -8.28% to -0.16%) and a non-statistically significant (*P* = 0.06) lower level of LDL (difference − 3.08%; 95% CI -6.16–0.1%). Other HbA1c and lipid profile associations with other food categories are available in supplementary Table S2. The modified healthful plant-based diet score in T2D patients was also assessed as a binary phenotype; CAD, stroke, (PAD and CKD (supplementary table S3). The associations between diet pattern in T2D patients and those comorbidities did not show an even trend for statistical significance, which may have been due to the small sample size for each of these binary phenotypes. However, in our study sample, the prevalence of CAD, stroke, PAD, and CKD in T2D patients were 11.3%, 6.2%, 3.3% and 8.4%, respectively. In addition, the highest quintiles vs. the lowest quintiles of modified healthful plant-based diet score are shown in Supplementary Table S4.

**Table 4:** Association of HbA1c, TC, TG, HDL, LDL with some individual food categories in patients with T2D, adjusting for age and sex

Food Categories	Variable	% change (95%CI) per score	*P*-Value
Butter Score	HbA1c (%)	4.44% (0.53– 8.5%)	0.03^#^
	TC (mg/dL)	1.78% (-3.17– 6.98%)	0.49
	TG (mg/dL)	-4.23% (-11.91%– 4.12%)	0.31
	HDL (mg/dL)	2.59% (-2.47– 7.9%)	0.32
	LDL (mg/dL)	-0.29% (-1.00%– 0.43%)	0.43
Fish Score	HbA1c (%)	-0.05% (-1.65%– 1.59%)	0.95
	TC (mg/dL)	1.6% (-0.59– 3.84%)	0.15
	TG (mg/dL)	3.09% (-0.61– 6.92%)	0.1
	HDL (mg/dL)	-1.79% (-3.91%– 0.37%)	0.1
	LDL (mg/dL)	3% (0.18– 5.9%)	0.04^#^
Whole Grains Score	HbA1c (%)	0.62% (-1.59– 2.88%)	0.58
	TC (mg/dL)	-0.88% (-3.85%– 2.18%)	0.57
	TG (mg/dL)	-4.2% (-8.96%– 0.8%)	0.1
	HDL (mg/dL)	3.69% (0.59– 6.88%)	0.02^#^
	LDL (mg/dL)	-2.41% (-6.13%– 1.45%)	0.22
Vegetable Score	HbA1c (%)	-0.3% (-2.41%– 1.85%)	0.78
	TC (mg/dL)	1.17% (-1.75– 4.18%)	0.43
	TG (mg/dL)	-3.91% (-8.5%– 0.92%)	0.11
	HDL (mg/dL)	4.2% (1.21– 7.27%)	0.01^#^
	LDL (mg/dL)	1.59% (-2.14– 5.46%)	0.41
Sugar Sweetened Beverages Score	HbA1c (%)	-1.11% (-2.91%– 0.74%)	0.24
	TC (mg/dL)	-1.91% (-4.37%– 0.61%)	0.14
	TG (mg/dL)	-4.31% (-8.28%– -0.16%)	0.04^#^
	HDL (mg/dL)	3.07% (0.49– 5.71%)	0.02^##^
	LDL (mg/dL)	-3.08% (-6.16%– 0.1%)	0.06

The higher the score, the healthier the amount of the food item consumed. ^#^= statistically significant at *p* < 0.05. HbA1c: glycated hemoglobin, TC: total cholesterol, TG: triglycerides. HDL: high density lipoprotein, LDL: low density lipoprotein

## Discussion

To the best of our knowledge, this is the first hospital-based study conducted to identify the dietary habits of T2D patients in the Eastern Province of Saudi Arabia. The Kingdom, with a population of over 20 million, has one of highest prevalence rates of T2D (≈ 18.7% of the total population), and the Eastern Province has reported the highest increase in the prevalence of T2D [19]. The rapid rate of increase of T2D in some areas of Saudi Arabia, which increased from 16% in 2005 to over 25% in 2011, is thought to be due to rapid lifestyle changes, such as diet and sedentary lifestyle, as well as adverse environmental factors [12].

In the present study, we assessed the pattern of dietary habits of Saudi T2D patients in the Eastern Province of Saudi Arabia and determined their effects on HbA1c level, lipid profile and health status. Dietary intervention and change of lifestyle are recommended as the initial treatment plan for this metabolic disorder.

High levels of HbA1c (> 7%) are reported to be associated with an increased risk of cardiovascular disease (CVD) and morbidity in patients with T2D [20]. Moreover, dyslipidemia is also known to increase the risk of disease complications in T2D patients. Consequently, both these conditions, T2D and CVD, are influenced by diet. Despite recommendations by the Saudi Diabetes Society to reduce fat intake, butter and hard margarine intake is still quite high. It has been reported that consumption of butter and margarine in the Gulf states and especially Saudi Arabia increases by 2% every year (Market analysis). Commercial butter is a known source of high saturated fatty acid (SFA) and low in mono and polyunsaturated fatty acid (PUFA) [20]. A meta-analysis conducted by Imamura et al. (2016), reported that replacing a diet with SFA with a diet high in PUFA significantly reduced HbA1c [21]. The present study data are in line with the study by Imamura et al., as the data show that T2D patients who consumed more butter had a significantly higher level of HbA1c. Although patients who consume soft margarine may have slightly lower HBA1c, hard margarines are known to contain a sizeable percentage of saturated and trans-fat and thus may have the same effects as butter in elevating HbA1c level. It has been reported that trans-fat affects lipid profile in an undesirable manner leading to no or slight change in LDL or HDL levels [22]. In the present study, modified hPDI scores for hard margarine and saturated fats were in the highest quintile in the minimum score criteria (supplementary table S2). Saturated fats, sweetened beverages and margarine scores were higher for our cohort due to the high frequency of intake of fast food and full fat dairy products. Many studies have suggested that increasing fish consumption is recommended for the body’s supply of omega-3 fatty acids and also to confer benefits for CVD risk reduction. We observed that serum TC and LDL-cholesterol were significantly higher among people on non-vegetarian diets. In our study, T2D patients who consumed, on average, a greater percentage of fish rather than meat had significantly higher LDL levels. Nevertheless, it should be noted that fish meat varies in polyunsaturated fatty acids levels [23–24]. PUFAs are reported to decrease serum TG levels and slightly reduce LDL levels, both of which were not shown in our data indicating that the fish type consumed by our patients may have a low level of lc-n3-PUFAs [22, 25]. Prawns and shrimps are very popular in the Eastern Province diet, and they are known to be rich in cholesterol [26].

Whole grain consumption has been associated with lowering LDL level in multiple studies [27–31]. However, an increase in HDL level from whole grain consumption has conflicting results and seems to be affected by the type of whole grain consumed [27–31]. Our data indicates that increased ingestion of whole grain is associated with a statistically non-significant increase in HDL level, and a decrease in LDL and TG levels. In non-T2D individuals, high consumption of vegetables was associated with lower TC and LDL [32]. In T2D individuals, a diet high in vegetables and fruit rich in flavonoid was associated with lower HbA1c level [33]. Unsuprisingly, T2D patients who followed a vegan diet had the best glycemic control and a lower LDL level [34]. Our results showed that T2D patients with a high vegetable diet score had a a non-significant increase in their HDL level but other lipid and glycemic control parameters were not significantly affected. This could be due to the type of vegetable being consumed, such as starch rich vegetables (e.g. squash, potatoes). It has also been reported that consumption of sugar sweetened beverages increased the level of TG and LDL and lowered the level of HDL [35–36]. This metabolic disturbance could be due to the high level of lipogenic sugar (fructose and sucrose) in these beverages [35, 37]. Our results show that adherence to a low consumption of sugar sweetened beverages was significantly associated with a lower level of TG and LDL and increased the level of HDL in Saudi T2D patients. High blood glucose levels over an extended period of time brought about by the consumption of sweetened beverages may result in the development of advanced glycated end-products, which cause diabetes-related macrovascular and microvascular changes [38]. Prevalence of CAD, PAD, stroke, and CKD was higher in our T2D group compared to the general population and was associated with a poor quality of life.

As the prevalence of T2D is reaching a pandemic state, dedicated awareness campaigns are being conducted by hospitals in the area to educate T2D patients and their families on the importance of a strict adherence to healthy dietary habits to improve their quality of life.

## Conclusion

The present study showed that adherence to a healthful plant-based diet, when compared to high glycemic index diet, is associated with a favorable outcome in glycemic control and lipid profile in T2D patients. Prior assessment of total diet quality may be beneficial when giving nutritional advice to T2D patients with the possibility of improving glycemic control and lipid profile.

### Electronic supplementary material

Below is the link to the electronic supplementary material.

Supplementary Material 1

Supplementary Material 2

Supplementary Material 3

Supplementary Material 4

Supplementary Material 5

## Data Availability

All data generated and analyzed during this study are included in this published article and its supplementary information files.

## Abbreviations

T2D: Type 2 Diabetes
DM: Diabetes
CAD: Coronary artery diseases
HbA1c: Glycated hemoglobin
MNT: Medical nutrition treatment
hPDI: Healthful Plant-based Diet Index
uPDI: Unhealthful plant-based diet index
7-d SQFFQ: 7-day semi-quantitative food frequency questionnaire
PAD: Peripheral artery disease
CKD: Chronic kidney disease
TC: Total cholesterol
TG: Triglycerides
LDL: Low density lipoprotein
HDL: High density lipoprotein
CVD: Cardiovascular disease
