# Supplementary material for: Dietary assessment of type 2 diabetic patients using healthful plant-based diet score in the Eastern Province of Saudi Arabia
Source: BMC Nutr. 2024 Feb 28;10:37. doi: 10.1186/s40795-024-00843-z (PMC10900584; doi:10.1186/s40795-024-00843-z)
Supplement: Supplementary file 1 — Supplementary Material 1 [file 40795_2024_843_MOESM1_ESM.pdf]

Supplementary Table S1A. Results of FFQ by each food items

Component	Items	Serving frequency category	N (%)	Serving frequency
Fat/Oil	Margarine	0	435 (83.2%)	1= once a week; 2= once or more a week; 3= every day
		1	52 (9.9%)	
		2	18 (3.4%)	
		3	18 (3.4%)	
	Butter	0	462 (88.3%)	
		1	36 (6.9%)	
		2	18 (3.4%)	
		3	7 (1.3%)	
	Vegetable Oil	0	46 (8.8%)	
		1	32 (6.1%)	
		2	26 (5%)	
		3	419 (80.1%)	
Dairy	Milk, full fat	0	398 (76.1%)	Cup per day
		1	84 (16.1%)	
		2	27 (5.2%)	
		3	9 (1.7%)	
		4	1 (0.2%)	
		5	4 (0.8%)	
	Milk, low fat	0	347 (66.4%)	
		1	120 (22.9%)	
		2	39 (7.5%)	
		3	11 (2.1%)	
		4	4 (0.8%)	
		5	2 (0.4%)	
	Milk, Skimmed	0	502 (96%)	
		1	15 (2.9%)	
		2	4 (0.8%)	
		3	2 (0.4%)	
	Cheese	0	90 (17.2%)	1=once a month; 2=1-3 times per month; 3=once a week; 4=2-4 times per week; 5=5-6 times per week; 6=once a day
		1	5 (1%)	
		2	20 (3.8%)	
		3	57 (10.9%)	
		4	142 (27.2%)	
		5	28 (5.4%)	
		6	181 (34.6%)	
	Yoghurt	0	80 (15.3%)	
		1	10 (1.9%)	

		2	28 (5.4%)	
		3	52 (9.9%)	
		4	154 (29.5%)	
		5	48 (9.2%)	
		6	151 (28.9%)	
Meat	Beef, Stewed	0	406 (77.6%)	
		1	30 (5.7%)	
		2	26 (5%)	
		3	29 (5.5%)	
		4	29 (5.5%)	
		5	1 (0.2%)	
		6	2 (0.4%)	
	Beef, Minced	0	414 (79.2%)	
		1	31 (5.9%)	
		2	35 (6.7%)	
		3	31 (5.9%)	
		4	11 (2.1%)	
		5	1 (0.2%)	
	Beef, Burgers	0	465 (88.9%)	
		1	28 (5.4%)	
		2	14 (2.7%)	
		3	11 (2.1%)	
		4	4 (0.8%)	
		5	1 (0.2%)	
	Lamb, Stewed	0	163 (31.2%)	
		1	47 (9%)	
		2	97 (18.6%)	
		3	92 (17.6%)	
		4	111 (21.2%)	
		5	4 (0.8%)	
		6	9 (1.7%)	
	Lamb, Minced	0	378 (72.3%)	
		1	19 (3.6%)	
		2	40 (7.7%)	
		3	42 (8%)	
		4	41 (7.8%)	
		5	1 (0.2%)	
		6	2 (0.4%)	
	Lamb, Burgers	0	452 (86.4%)	
		1	20 (3.8%)	
		2	17 (3.3%)	

		3	18 (3.4%)	
		4	12 (2.3%)	
		5	2 (0.4%)	
		6	2 (0.4%)	
	Chicken, Stewed	0	149 (28.5%)	
		1	9 (1.7%)	
		2	14 (2.7%)	
		3	37 (7.1%)	
		4	163 (31.2%)	
		5	97 (18.6%)	
		6	54 (10.3%)	
	Chicken, Minced	0	366 (70%)	
		1	9 (1.7%)	
		2	11 (2.1%)	
		3	13 (2.5%)	
		4	74 (14.2%)	
		5	40 (7.7%)	
		6	10 (1.9%)	
	Chicken, Burgers	0	461 (88.2%)	
		1	7 (1.3%)	
		2	8 (1.5%)	
		3	14 (2.7%)	
		4	17 (3.3%)	
		5	4 (0.8%)	
		6	12 (2.3%)	
Fish	Fish, Grilled	0	292 (55.8%)	
		1	29 (5.5%)	
		2	54 (10.3%)	
		3	68 (13%)	
		4	71 (13.6%)	
		5	7 (1.3%)	
		6	2 (0.4%)	
	Fish, Fried	0	215 (41.1%)	
		1	37 (7.1%)	
		2	81 (15.5%)	
		3	88 (16.8%)	
		4	96 (18.4%)	
		5	5 (1%)	
		6	1 (0.2%)	
Potatoes	Potatoes	0	182 (34.8%)	
		1	24 (4.6%)	

		2	36 (6.9%)	
		3	107 (20.5%)	
		4	138 (26.4%)	
		5	13 (2.5%)	
		6	23 (4.4%)	
Vegetables	Salad	0	40 (7.7%)	
		1	3 (0.6%)	
		2	10 (1.9%)	
		3	34 (6.5%)	
		4	92 (17.6%)	
		5	47 (9%)	
		6	297 (56.8%)	
Whole grains	Brown bread	0	211 (40.3%)	1=once a month; 2=1-3 per month ,3=once a week; 4=2-4 per week, 5=5-6 per week, 6=one a day; 8=4-5 per day, 9= 6 per day
		1	4 (0.8%)	
		2	4 (0.8%)	
		3	4 (0.8%)	
		4	110 (21%)	
		5	5 (1%)	
		6	180 (34.4%)	
		8	3 (0.6%)	
		9	2 (0.4%)	
Refined grains	White bread	0	270 (51.6%)	
		1	5 (1%)	
		2	2 (0.4%)	
		3	6 (1.2%)	
		4	72 (13.8%)	
		5	6 (1.2%)	
		6	159 (30.4%)	
		8	1 (0.2%)	
		9	2 (0.4%)	
	Porridge	0	410 (78.4%)	1=once a month; 2=1-3 times per month; 3=once a week; 4=2-4 times per week; 5=5-6 times per week; 6=once a day
		1	55 (10.5%)	
		2	47 (9%)	
		3	6 (1.2%)	
		4	3 (0.6%)	
		6	2 (0.4%)	
	Cornflakes	0	418 (79.9%)	
		1	36 (6.9%)	
		2	26 (5%)	
		3	19 (3.6%)	
		4	14 (2.7%)	

		6	10 (1.7%)	
	Pizza	0	246 (47%)	
		1	83 (15.9%)	
		2	115 (22%)	
		3	44 (8.4%)	
		4	28 (5.4%)	
		5	3 (0.6%)	
		6	4 (0.8%)	
	Pasta	0	120 (22.9%)	
		1	45 (8.6%)	
		2	138 (26.4%)	
		3	135 (25.8%)	
		4	77 (14.7%)	
		5	1 (0.2%)	
		6	7 (1.3%)	
	Rice	0	25 (4.8%)	
		1	4 (0.8%)	
		2	18 (3.4%)	
		3	34 (6.5%)	
		4	160 (30.6%)	
		5	62 (11.9%)	
		6	220 (42.1%)	
Fruits	Tinned fruit	0	456 (87.2%)	
		1	17 (3.3%)	
		2	7 (1.3%)	
		3	7 (1.3%)	
		4	11 (2.1%)	
		5	6 (1.2%)	
		6	19 (3.6%)	
	Peaches, plums	0	188 (36%)	
		1	13 (2.5%)	
		2	41 (7.8%)	
		3	39 (7.5%)	
		4	97 (18.6%)	
		5	26 (5%)	
		6	119 (22.8%)	
	Grapes	0	149 (28.5%)	
		1	16 (3.1%)	
		2	45 (8.6%)	
		3	46 (8.8%)	
		4	118 (22.6%)	

		5	26 (5%)	
		6	123 (23.5%)	
	Melon	0	160 (30.6%)	
		1	19 (3.6%)	
		2	40 (7.7%)	
		3	43 (8.2%)	
		4	103 (19.7%)	
		5	22 (4.2%)	
		6	136 (26%)	
	Bananas	0	74 (14.2%)	
		1	7 (1.3%)	
		2	25 (4.8%)	
		3	54 (10.3%)	
		4	134 (25.6%)	
		5	33 (6.3%)	
		6	196 (37.5%)	
	Apples	0	47 (9%)	
		1	5 (1%)	
		2	21 (4%)	
		3	50 (9.6%)	
		4	143 (27.3%)	
		5	43 (8.2%)	
		6	214 (40.9%)	
	Oranges	0	70 (13.4%)	
		1	8 (1.5%)	
		2	23 (4.4%)	
		3	47 (9%)	
		4	137 (26.2%)	
		5	34 (6.5%)	
		6	204 (39%)	
Fruit juices	Juice	0	151 (28.9%)	
		1	18 (3.4%)	
		2	28 (5.4%)	
		3	56 (10.7%)	
		4	141 (27%)	
		5	27 (5.2%)	
		6	102 (19.5%)	
Sweets and desserts	Chocolate	0	231 (44.2%)	
		1	50 (9.6%)	
		2	71 (13.6%)	
		3	65 (12.4%)	

		4	62 (11.9%)	
		5	5 (1%)	
		6	39 (7.5%)	
	Cakes	0	160 (30.6%)	
		1	67 (12.8%)	
		2	102 (19.5%)	
		3	100 (19.1%)	
		4	71 (13.6%)	
		5	8 (1.5%)	
		6	15 (2.9%)	
Sugar sweetened beverages	Sweetened Tea	0	352 (67.3%)	
		1	20 (3.8%)	
		2	1 (0.2%)	
		3	15 (2.9%)	
		4	67 (12.8%)	
		5	1 (0.2%)	
		6	67 (12.8%)	
	Cola	0	343 (65.6%)	
		1	31 (5.9%)	
		2	23 (4.4%)	
		3	44 (8.4%)	
		4	39 (7.5%)	
		5	4 (0.8%)	
		6	39 (7.5%)	
Tea and coffee	Coffee	0	85 (16.3%)	
		1	7 (1.3%)	
		2	9 (1.7%)	
		3	31 (5.9%)	
		4	191 (36.5%)	
		5	9 (1.7%)	
		6	191 (36.5%)	
	Tea	0	74 (14.2%)	
		1	10 (1.9%)	
		2	6 (1.2%)	
		3	17 (3.3%)	
		4	207 (39.6%)	
		5	6 (1.2%)	
		6	203 (38.8%)	
