# Supplementary material for: Dietary assessment of type 2 diabetic patients using healthful plant-based diet score in the Eastern Province of Saudi Arabia
Source: BMC Nutr. 2024 Feb 28;10:37. doi: 10.1186/s40795-024-00843-z (PMC10900584; doi:10.1186/s40795-024-00843-z)
Supplement: Supplementary file 2 — Supplementary Material 2 [file 40795_2024_843_MOESM2_ESM.pdf]

Supplementary Table S1B: Modified Healthful Plant-based Diet Index components and criteria for scoring

**Component (serving frequency; N (%))**	**Minimum score (least healthy)**				**Maximum score (healthiest)**
	**1**	**2**	**3**	**4**	**5**
Healthy Plant Food Groups					
Whole grains (Bread, brown)*#	0; 211 (40%)	(0,4]; 122 (23%)	(4,6]; 185 (35%)	(6,9]; 5 (1%)	--
Fruits	[0,12]; 116 (22%)	(12,18]; 113 (22%)	(18,24]; 118 (23%)	(24,32.6]; 71 (14%)	(32.6,42]; 105 (20%)
Vegetables (salad)‡	[0,4]; 179 (34%)	(4,5]; 47 (9%)	6; 297 (57%)		
Vegetable oils##	0; 46 (9%)	1; 32 (6%)	2; 26 (5%)	3; 419 (80%)	--
Tea & Coffee#	[0,6]; 155 (30%)	(6,8]; 134 (25%)	(8,10]; 123 (24%)	(10,12]; 111 (21%)	--
Unhealthy Plant Food Groups					
Fruit juices	(5,6]; 102 (20%)	(4,5]; 27 (5%)	(3,4]; 141 (27%)	(0,3]; 102 (20%)	0; 151 (29%)
Refined grains*	(15,24]; 93 (18%)	(12,15]; 114 (22%)	(10,12]; 76 (15%)	(7,10]; 127 (24%)	[0,7]; 113 (22%)
Potatoes	(4,6]; 36 (7%)	(3,4]; 138 (26%)	(2,3]; 107 (20%)	(0,2]; 60 (11%)	0; 182 (35%)
Sugar sweetened beverages (sweentened tea, cola)#	(6,12]; 59 (11%)	(3,6]; 131 (25%)	(0,3]; 77 (15%)	0; 256 (49%)	
Sweets and desserts (chocolate, cake)	(6,12]; 85 (16%)	(4,6]; 86 (16%)	(2,4]; 130 (25%)	(0,2]; 96 (18%)	0; 126 (24%)
Animal Food Groups					
Animal fat (Butter)##	3; 7 (1%)	2; 18 (3%)	1; 36 (7%)	0; 462 (88%)	--
Dairy	(12,16]; 105 (20%)	(10,12]; 61 (12%)	(8,10]; 114 (22%)	(6,8]; 103 (20%)	[0,6]; 140 (27%)
Fish or seafood	(4,12]; 51 (10%)	(3,4]; 136 (26%)	(2,3]; 125 (24%)	2; 120 (23%)	[0,2); 91 (17%)
Meat (beef, lamb, chicken)	(11,39]; 90 (17%)	(8,11]; 118 (23%)	8; 108 (21%)	(6,8); 80 (15%)	[0,6]; 127 (24%)
Trans Fat (Margarine)##	3; 18 (3%)	2; 18 (3%)	1; 52 (10%)	0; 435 (83%)	--

*: 1=once a month; 2=1-3 per month ,3=once a week; 4=2-4 per week, 5=5-6 per week, 6=one a day; 8

#: Only 4 categories were recorded; ‡: Only 3 category we

##: 0: None; 1= once a week; 2= once or more a week; 3=
