# Supplementary material for: Dietary assessment of type 2 diabetic patients using healthful plant-based diet score in the Eastern Province of Saudi Arabia
Source: BMC Nutr. 2024 Feb 28;10:37. doi: 10.1186/s40795-024-00843-z (PMC10900584; doi:10.1186/s40795-024-00843-z)
Supplement: Supplementary file 3 — Supplementary Material 3 [file 40795_2024_843_MOESM3_ESM.pdf]

Supplementary Table S2: Association of HbA1c, TC, TG, HDL, LDL with some individual food categories in patients with diabetes mellites, adjusting for age and sex

Category	Variable	% change (95%CI) per quintile	P-Value
Margarine Score	HbA1c	0.09% (-2.81% – 3.07%)	0.95
	TC	-0.99% (-4.86% – 3.04%)	0.62
	TG	-0.61% (-7.05% – 6.27%)	0.86
	HDL	-1.82% (-5.66% – 2.18%)	0.37
	LDL	-1.01% (-5.92% – 4.16%)	0.7
Vegetable Oil Score	HbA1c	-1.34% (-3.37% – 0.74%)	0.2
	TC	1.09% (-1.78% – 4.05%)	0.46
	TG	-0.92% (-5.6% – 3.99%)	0.71
	HDL	2.71% (-0.23% – 5.73%)	0.07
	LDL	1.33% (-2.36% – 5.16%)	0.48
Dairy Score	HbA1c	0.34% (-1% – 1.7%)	0.62
	TC	-0.7% (-2.53% – 1.18%)	0.46
	TG	-1.07% (-4.12% – 2.07%)	0.5
	HDL	-0.99% (-2.83% – 0.87%)	0.29
	LDL	-0.56% (-2.9% – 1.84%)	0.65
Animal Meat (excluding Fish) Score	HbA1c	-0.76% (-2.14% – 0.64%)	0.29
	TC	0.24% (-1.67% – 2.18%)	0.81
	TG	-1.75% (-4.86% – 1.47%)	0.28
	HDL	0.71% (-1.21% – 2.67%)	0.47
	LDL	0.91% (-1.54% – 3.41%)	0.47
Potato Score	HbA1c	0.21% (-1.21% – 1.66%)	0.77
	TC	-1% (-2.93% – 0.97%)	0.32
	TG	-0.98% (-4.2% – 2.34%)	0.56
	HDL	-0.93% (-2.86% – 1.04%)	0.35
	LDL	-1.8% (-4.24% – 0.69%)	0.16
Refined Grains Score	HbA1c	-0.57% (-1.97% – 0.84%)	0.43
	TC	-0.31% (-2.24% – 1.65%)	0.75
	TG	-2.66% (-5.78% – 0.56%)	0.1
	HDL	1.56% (-0.4% – 3.55%)	0.12
	LDL	-1.2% (-3.62% – 1.28%)	0.34
Fruits Score	HbA1c	-0.9% (-2.25% – 0.46%)	0.19
	TC	-0.26% (-2.13% – 1.65%)	0.79
	TG	-1% (-4.09% – 2.2%)	0.54
	HDL	1.65% (-0.25% – 3.59%)	0.09
	LDL	-1.32% (-3.68% – 1.09%)	0.28
Sweets & Desserts Score	HbA1c	1.28% (-0.18% – 2.75%)	0.08
	TC	-1.21% (-3.16% – 0.77%)	0.23
	TG	-0.33% (-3.59% – 3.05%)	0.85
	HDL	-1.7% (-3.64% – 0.27%)	0.09
	LDL	-0.38% (-2.88% – 2.18%)	0.77
Tea & Coffee Score	HbA1c	-0.22% (-1.98% – 1.57%)	0.81
	TC	0.64% (-1.77% – 3.12%)	0.6
	TG	-1.52% (-5.45% – 2.58%)	0.46
	HDL	2.15% (-0.3% – 4.66%)	0.09
	LDL	1.92% (-1.18% – 5.13%)	0.23
