# Supplementary material for: Dietary assessment of type 2 diabetic patients using healthful plant-based diet score in the Eastern Province of Saudi Arabia
Source: BMC Nutr. 2024 Feb 28;10:37. doi: 10.1186/s40795-024-00843-z (PMC10900584; doi:10.1186/s40795-024-00843-z)
Supplement: Supplementary file 4 — Supplementary Material 4 [file 40795_2024_843_MOESM4_ESM.pdf]

Supplementary Table S3. Association of CAD/Stroke/PAD/CKD with Modified Healthful Plant-based Diet Score (as Quintiles) Among DM Patients, Adjusting for Age and Sex

Phenotype	Number of Cases	Number of Controls	OR (95%CI)	P-Value	
CAD	55	432	1.06 (0.87 – 1.30)	0.56	
Stroke	30	457	1.09 (0.84 – 1.42)	0.52	
PAD	16	471	1.17 (0.82 – 1.66)	0.39	
CAD	41	446	1.03 (0.82 – 1.30)	0.8	
Two or more comorbidities[#]	22	487	1.23 (0.90 – 1.68)	0.2	
Two or more comorbidities[##]	22	370	1.20 (0.87 – 1.64)	0.26	
* Scores were classified into quintiles					
[#]: Control group: participants with one or no comorbidities					
[##]: Control group: participants with no comorbidities					
