# Supplementary material for: Dietary assessment of type 2 diabetic patients using healthful plant-based diet score in the Eastern Province of Saudi Arabia
Source: BMC Nutr. 2024 Feb 28;10:37. doi: 10.1186/s40795-024-00843-z (PMC10900584; doi:10.1186/s40795-024-00843-z)
Supplement: Supplementary file 5 — Supplementary Material 5 [file 40795_2024_843_MOESM5_ESM.pdf]

Supplementary Table S4. Association of CAD/Stroke/PAD/CKD with Modified Healthful Plant-based Diet Score (as Quintiles) Among DM Patients: Comparing patients in Higest vs. Lowest Diet Score Quintiles, Adjusting for Age and Sex

Phenotype	Number of Cases	Number of Controls	OR (95%CI)	P-Value
CAD	19	182	0.99 (0.36 – 2.75)	0.99
Stroke	13	110	1.47 (0.46 – 4.73)	0.52
PAD	6	195	3.09 (0.53 – 18.0)	0.21
CAD	15	186	1.07 (0.35 – 3.23)	0.91
Two or more comorbidities[#]	10	191	2.02 (0.51 – 7.94)	0.32
Two or more comorbidities[##]	10	160	2.07 (0.51 – 8.36)	0.31

* Scores were classified into quintiles

[#]: Control group: participants with one or none comorbidities

[##]: Control group: participants with no comorbidities
